# Cytogenetic monoclonality in multifocal uroepithelial carcinomas: evidence of intraluminal tumour seeding

**DOI:** 10.1038/sj.bjc.6690643

**Published:** 1999-09

**Authors:** I Fadl-Elmula, L Gorunova, N Mandahl, P Elfving, R Lundgren, F Mitelman, S Heim

**Affiliations:** Departments of Clinical Genetics and Urology, University Hospital, Lund, S-221 85, Sweden; Department of Urology, Helsingborg Hospital, Helsingborg, S-251 87, Sweden; Department of Genetics, The Norwegian Radium Hospital and Institute for Cancer Research, Oslo, N-0310, Norway

**Keywords:** cytogenetics, multifocal uroepithelial carcinomas, monoclonality, intraluminal seeding

## Abstract

Twenty-one multifocal urinary tract transitional cell carcinomas, mostly bladder tumours, from a total of six patients were processed for cytogenetic analysis after short-term culturing of the tumour cells. Karyotypically related, often identical, cytogenetically complex clones were found in all informative tumours from each case, including the recurrent tumours. Rearrangement of chromosome 9, leading to loss of material from the short and/or the long arm, was seen in all cases, indicating that this is an early, pathogenetically important event in transitional cell carcinogenesis. The presence of related clones with great karyotypic similarity in anatomically distinct tumours from the same bladder indicates that multifocal uroepithelial tumours have a monoclonal origin and arise via intraluminal seeding of viable cancer cells shed from the original tumour. Later lesions may develop also from cells shed from the so called second primary tumours. The relatively complex karyotypes seen in all lesions from most cases argue that the seeding of tumour cells is a late event that succeeds the acquisition by them of multiple secondary genetic abnormalities. © 1999 Cancer Research Campaign


*British Journal of Cancer* (1999) **81**(1), 6–12
© 1999 Cancer Research Campaign
Article no. bjoc.1999.0643

### Cytogenetic monoclonality in multifocal uroepithelial carcinomas: evidence of intraluminal tumour seeding

**I Fadl-Elmula[1,3], L Gorunova[1], N Mandahl[1], P Elfving[2], R Lundgren[3], F Mitelman[1] and S Heim[4]**

Departments of [1]Clinical Genetics and [2]Urology, University Hospital, S-221 85 Lund, Sweden; [3]Department of Urology, Helsingborg Hospital, S-251 87 Helsingborg, Sweden; [4]Department of Genetics, The Norwegian Radium Hospital and Institute for Cancer Research, N-0310 Oslo, Norway

**Summary** Twenty-one multifocal urinary tract transitional cell carcinomas, mostly bladder tumours, from a total of six patients were processed for cytogenetic analysis after short-term culturing of the tumour cells. Karyotypically related, often identical, cytogenetically complex clones were found in all informative tumours from each case, including the recurrent tumours. Rearrangement of chromosome 9, leading to loss of material from the short and/or the long arm, was seen in all cases, indicating that this is an early, pathogenetically important event in transitional cell carcinogenesis. The presence of related clones with great karyotypic similarity in anatomically distinct tumours from the same bladder indicates that multifocal uroepithelial tumours have a monoclonal origin and arise via intraluminal seeding of viable cancer cells shed from the original tumour. Later lesions may develop also from cells shed from the so called second primary tumours. The relatively complex karyotypes seen in all lesions from most cases argue that the seeding of tumour cells is a late event that succeeds the acquisition by them of multiple secondary genetic abnormalities.

**Keywords:** cytogenetics; multifocal uroepithelial carcinomas; monoclonality; intraluminal seeding

Bladder cancer (BC) is the fourth most common cancer in men and the seventh most common cancer in women (Lynch et al, 1995). In Europe and the USA transitional cell carcinoma (TCC) dominates and accounts for 95% of all BC (Parker et al, 1996), making it the 12th leading cause of cancer death (Heney et al, 1983). Around 30% of urinary tract TCCs are found as multiple tumours at the time of diagnosis (Kiemeney et al, 1993). Although 70% of newly diagnosed bladder TCCs are superficial, i.e. the tumour is confined to the lamina propria, the disease represents a major therapeutic challenge and a continuous threat to the patients since the tumours recur after transurethral resection of the primary in more than 70% of the cases (Heney et al, 1983; Thompson et al, 1993). In addition, progression to more advanced stages and/or grades occurs in 16–25% of the cases (Prout et al, 1992).

Implicit in the clinical observations above are several questions concerning the clonal origin of multifocal uroepithelial carcinomas and the mechanisms that are responsible for the local as well as distant dissemination of cancer cells (recurrences and metastases respectively). Many investigators, perhaps motivated by the polychronotopicity of BC and the dysplastic changes that are usually found in the mucosa surrounding bladder tumours (Wolf et al, 1982), have drawn the conclusion that a field defect must be present (Richie et al, 1989). According to this field disease theory, the entire epithelium is tumour prone in the sense that multiple polyclonal primary lesions are likely to emerge from it, either synchronously or metachronously (Yao et al, 1994). The alternative, monoclonal view presupposes a common clonal origin to all uroepithelial tumours, even the multifocal ones, implying that these macroscopically distinct lesions develop as the result of intraluminal seeding of cancer cells shed from the original tumour. Support for the monoclonal hypothesis has come from analyses of X-chromosome inactivation in the tumour cells of female patients (Sidransky et al, 1992). Other molecular genetic studies, mainly relying on the precise identification of *TP53* mutations as clonal markers in multifocal uroepithelial tumours (Habuchi et al, 1993; Chern et al, 1996; Xu et al, 1996), have provided evidence pointing in the same direction. However, since the tumours investigated in these studies were rather advanced as far as stage and grade are concerned, they do not necessarily yield information about the early genetic events in disease progression and dissemination. No corresponding cytogenetic study has been performed, in spite of the fact that this technique is particularly well suited to assess the clonal nature of neoplasms (Heim, 1992). We therefore decided to examine multifocal uroepithelial (mostly bladder) tumours using chromosome banding analysis after short-term culturing of the tumour cells.

### MATERIALS AND METHODS

Samples from a total of 21 macroscopically distinct uroepithelial tumour lesions (12 primary bladder tumours, two primary ureteral tumours, six recurrent bladder tumours and one recurrent tumour located in the prostatic urethra) were obtained from a total of six patients, all with multifocal disease.

**Patient 1** was a 54-year-old man who was first seen because of macroscopic haematuria. On cystoscopic examination, four papillary bladder tumours were noted (BT1–BT4) (Figure 1). A transurethral resection of the tumours was performed and a specimen for cytogenetic analysis was obtained from each tumour.

**Patient 2** was a 75-year-old woman who was admitted to hospital with a tentative diagnosis of pericarditis. The patient had no previous history of urological problems but, because an abdominal

*Received 13 October 1998*
*Revised 16 February 1999*
*Accepted 15 March 1999*

*Correspondence to:* I Fadl-Elmula, Department of Clinical Genetics, University Hospital, S-221 85 Lund, Sweden

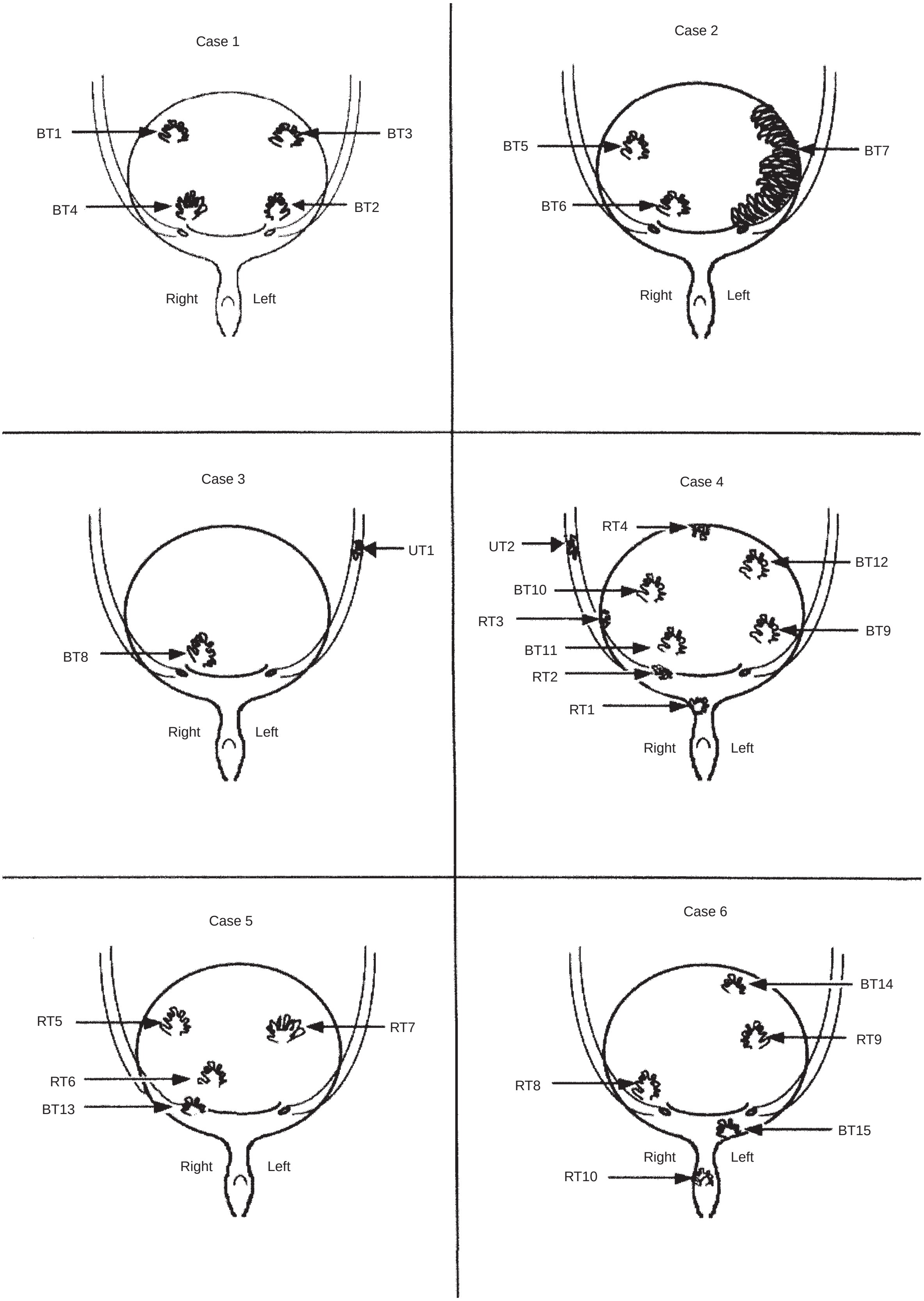

**Figure 1** Schematic illustration of the anatomical distribution of the 27 multifocal primary and recurrent uroepithelial carcinomas from the six patients. BT, bladder tumour; UT, ureteral tumour; RT, recurrent tumour ultrasound examination indicated the presence of multiple bladder tumours, he was transferred to the urology department for assessment and further management. On cystoscopic examination, three bladder tumours were found (BT5–BT7) (Figure 1), and a specimen from each was obtained after transurethral resection of the tumours.

**Patient 3** was a 77-year-old man who at initial presentation had two multicentric uroepithelial tumours, one in the bladder (BT8) and the other in the left distal ureter (UT1) (Figure 1). A sample from the bladder tumour was obtained by transurethral resection, whereas the ureteral tumour was removed by segmental ureteral resection followed by ureter reimplantation.

**Patient 4** was a 64-year-old man who had a total of five uroepithelial tumours, four in the bladder (BT9–BT12) and one in the upper right ureter (UT2) (Figure 1). Samples for cytogenetic analysis were obtained after transvesical resection of the bladder tumours, whereas the ureteral tumour was removed by nephroureterectomy. Three months later, a cystoscopy control showed four new papillary tumours in the bladder (RT1–RT4) (Figure 1). A specimen for cytogenetic analysis was obtained by transurethral resection of the tumour (RT1) located in the bladder neck (Figure 1).

**Patient 5** was a 75-year-old woman in whom a primary bladder tumour (BT13) (Figure 1) was detected in July 1997 and recurrent disease in October 1997. On each occasion, the tumours were endoscopically removed without any adjuvant treatment. In February 1998, the patient developed three multifocal recurrent bladder tumours (RT5–RT7) (Figure 1). These were removed by transurethral resection and a specimen from each was obtained for cytogenetic analysis.

**Patient 6** was a 74-year-old man with a history of bladder tumours (BT14 and BT15) (Figure 1) since January 1993. In spite of surgical treatment, by 1996 he had developed six recurrences. Adjuvant treatment with bacille Calmette-Guérin (BCG) was therefore added to the surgical measures, and when this proved to be of limited effect, intravesical epirubicine treatment was given. The patient nevertheless developed a recurrence in July 1997, and in March 1998, two tumours in the bladder and one in the prostatic urethra (RT8–RT10) were found (Figure 1). The tumours were endoscopically removed and a specimen from each obtained for cytogenetic analysis.

In all cases, great care was taken to avoid contamination or mixing when the macroscopically separate tumours were sampled or removed. All specimens were sent in separate containers to the cytogenetics laboratory. Similar care was taken during the histological examination of each tumour. Grading and staging were done according to WHO (1973) criteria and the UICC (1978) tumour-node-metastasis (TNM) classification system respectively.

All samples intended for cytogenetic analysis were processed separately. Short-term culturing and cytogenetic analysis were done as described by Fadl-Elmula et al (1998). In brief, the specimens were disaggregated mechanically and enzymatically using collagenase II. The resulting cells and cell clumps were washed three times in RPMI-1640 medium, plated out in vitrogen-coated flasks and fed a modified Dulbecco's modified Eagle's medium (DMEM)–F12 (1:1) medium. All cultures were harvested within 10 days. Colcemid was added 3–4 h before harvesting and the cells were detached by trypsinization. After hypotonic shock in 0.05 M potassium chloride, the cells were fixed three times in methanol:acetic acid (3:1). G-banding was obtained with Wright's stain. Between 25 and 100 metaphase cells were analysed for each tumour. The clonality criteria and the karyotype descriptions were according to the ISCN (1995) recommendation.

### RESULTS

The clinical and cytogenetic findings are summarized in Table 1 and Figures 1–3. The attempt to establish short-term cultures was successful in all but samples BT3 and BT4 from patient 1, which were hence uninformative. The cultures from all other samples revealed clonal chromosomal abnormalities. Apart from chromosome 15, all chromosomes were involved in numerical and/or structural rearrangements at least once. The most consistent was rearrangement of chromosome 9, leading to loss of all or parts of 9p and/or 9q in all examined tumours, followed by rearrangement of chromosomes 1, 3, 7, 8 and 11 in three cases each. Rearrangements of chromosome 17 resulting in loss of the short arm were seen in cases 5 and 6, and in tumour BT7 from case 2. Except for case 6, in which hypotetraploid clones were seen, all stemlines were pseudo- or near-diploid; an additional duplicated hypertetraploid clone was seen in the tumours from patient 4. In all patients, the various tumours were cytogenetically identical or, at least, clonally related, the cytogenetic differences being restricted to one to three aberrations.

### DISCUSSION

Because 70% of bladder carcinomas are superficial at diagnosis (Thompson et al, 1993), surgical cure ought to be attainable in at least these patients. The actual clinical course in BC is nevertheless remarkably heterogeneous, with as many as 60% of patients with superficial disease having recurrence(s) after transurethral resection of the primary tumour(s) (Fitzpatrick et al, 1986). Good prognostic markers and a better understanding of the pathogenic mechanisms behind local and distant disease spreading are therefore urgently needed. Such information might help design new therapeutic strategies geared towards the prevention of tumour recurrence specifically and, in general, a more aggressive approach in progression-prone cases.

There are three mechanisms that have been implicated in the frequent multicentricity and high recurrence rate characteristic of BC (Schmitz-Dräger et al, 1996): implantation of cancer cells shed from the primary tumour, growth of new carcinoma(s) at remote sites, and regrowth of an incompletely resected primary tumour. Against the regrowth hypothesis speaks the fact that the new tumour almost always appears at a site different from the primary (Harris et al, 1992). In addition, biopsying of the scar area after removal of the primary tumour has failed to demonstrate any post-resection residual cancer, especially in cases with superficial disease (Badalament et al, 1996). The consistent cytogenetic monoclonality among macroscopically distinct lesions found in our study therefore speaks strongly in favour of the shedding and seeding hypothesis, since the simultaneous growth of multiple, pathogenically independent tumours is well-right irreconcilable with the observed monoclonality. This conclusion is in complete agreement with the impressions left by previous findings obtained by molecular genetic techniques (Sidransky et al, 1992; Habuchi et al, 1993; Chern et al, 1996; Xu et al, 1996).

All examined tumours showed cytogenetic aberrations involving the short and/or long arm of chromosome 9, and indeed the importance of chromosome 9 rearrangement as an early, presumably primary, event in BC tumorigenesis is widely accepted (Miyao et al, 1993; Gibas et al, 1997). The ubiquity of chromosome 9 changes in our series may be contrasted with the absence

 © 1999 Cancer Research Campaign

**Table 1** Clinical and cytogenetic data on 21 multifocal uroepithelial carcinomas

| Case | Sex/Age | Tumour/Site | Stage/Grade | Karyotype |
|---|---|---|---|---|
| 1 | M/54 | BT1/Bladder | Ta/G2 | 47,X,-Y,del(2)(q21q31),t(3;5)(q27;q31),del(5)(q11q13),+7,–9,+r,+mar[50] |
| | | BT2/Bladder | T1/G2 | 46,X,-Y,del(2)(q21q31),t(3;5)(q27;q31),del(5)(q11q13),+7,–9,der(9;13)(q10;q10),+13,+r[45] |
| 2 | F/75 | BT5/Bladder | Ta/G1–2 | 46,X,t(X;9)(p11;p11),del(1)(p11),+del(1)(p11),del(7)(q11),i(8)(q10),add(9)(q12),der(11)t(7;11)(q11;p15)del(11)(q12),der(13)t(1;13)(p11;p11),del(13)(q13q22),–14[100] |
| | | BT6/Bladder | Ta/G1–2 | 46,X,t(X;9)(p11;p11),del(1)(p11),+del(1)(p11),del(7)(q11),i(8)(q10),add(9)(q12),der(11)t(7;11)(q11;p15)del(11)(q12),der(13)t(1;13)(p11;p11),del(13)(q13q22),–14[100] |
| | | BT7/Bladder | T1/G3 | 46,X,t(X;9)(p11;p11),del(1)(p11),+del(1)(p11),del(7)(q11),add(9)(q12),der(11)t(7;11)(q11;p15)del(11)(q12),der(13)t(1;13)(p11;p11),–14[30]/46,X,t(X;9)(p11;p11),del(1)(p11),del(1)(p11),del(7)(q11),i(8)(q10),add(9)(q12),der(11)t(7;11)(q11;p15)del(11)(q12),der(13)t(1;13)(p11;p11),del(13)(q13q22),–14[40]/46,X,t(X;9)(p11;p11),del(1)(p11),+del(1)(p11),del(7)(q11),i(8)(q10),add(9)(q12),der(11)t(7;11)(q11;p15)del(11)(q12),der(13)t(1;13)(p11;p11),del(13)(q13q22),–14,del(17)(p11)[26] |
| 3 | M/77 | UT1/Ureter | Ta/G1–2 | 46,XY,+1,der(1;14)(q10;q10),del(3)(q27),add(4)(q34),–9,+mar[30] |
| | | BT8/Bladder | Ta/G1 | 45,XY,+1,der(1;14)(q10;q10),del(3)(q27),add(4)(q34),–9[25] |
| 4 | M/64 | UT2/Ureter | T1/G2 | 48–50,XY,del(3)(p13p23),+8,add(8)(p11)×2,add(9)(p11),del(11)(q13q21),i(11)(q10),add(14)(q21),del(16)(q22),–18,add(20)(q11),+3–4mar[cp27]/9–100,idemx2[cp10]/46,XY[35] |
| | | BT9/Bladder | T1/G2 | 48–50,XY,del(3)(p13p23),+8,add(8)(p11)×2,add(9)(p11),del(11)(q13q21),i(11)(q10),del(16)(q22),–18,+19,+3–4mar[cp60]/96–100,idemx2[cp20]/46,XY[25] |
| | | BT10/Bladder | T1/G2 | 48–50,XY,del(3)(p13p23),+8,add(8)(p11)×2,–9,del(11)(q13q21),i(11)(q10),der(18)t(1;18)(q21;q21),+3–4mar[cp23]/96–100,idemx2[cp9]/46,XY[15] |
| | | BT11/Bladder | T1/G2 | 48–50,XY,del(3)(p13p23),+8,add(8)(p11)×2,add(9)(p11),del(11)(q13q21),i(11)(q10),del(16)(q22),–18,+3–4mar[cp37]/96–100,idemx2[cp6]/46,XY[14] |
| | | BT12/Bladder | T1/G2 | 48–50,XY,del(3)(p13p23),+8,add(8)(p11)×2,add(9)(p11),del(11)(q13q21),i(11)(q10),add(14)(q21),del(16)(q22),–18,+3–4mar[cp22]/96–100,idemx2[cp11]/46,XY[24] |
| | | RT1/Bladder | T1/G2 | 48–50,XY,del(3)(p13p23),+8,add(8)(p11)×2,add(9)(p11),del(11)(q13q21),i(11)(q10),add(14)(q21),del(16)(q22),–18,+3–4mar[cp44]/96–100,idem×2[cp10]/46,XY[20] |
| 5 | 75/F | RT5/Bladder | T1/G2 | 43,XX,–9,del(10)(q22q24),–11,der(12;17)(q10;q10)[25] |
| | | RT6/Bladder | T1/G2 | 43,XX,–9,del(10)(q22q24),–11,der(12;17)(q10;q10)[25] |
| | | RT7/Bladder | T1/G2 | 43,XX,–9,del(10)(q22q24),–11,der(12;17)(q10;q10)[25] |
| 6 | 74/M | RT8/Bladder | Ta/G2 | 88–93,XXYY,add(6)(p21),add(8)(p11),del(8)(p22),–9,add(16)(p11),der(17)t(1;17)(q12;p11),+mar[cp28] |
| | | RT9/Bladder | Ta/G2 | 89–94,XXYY,add(6)(p21),add(8)(p11),del(8)(p22),–9,add(16)(p11),der(17)t(1;17)(q12;p11),–1+1–2mar[cp25] |
| | | RT10/Bladder | Ta/G2 | 89–93,XXYY,add(6)(p21),add(8)(p11),del(8)(p22),–9,add(16)(p11),der(17)t(1;17)(q12;p11),+mar[cp30] |

© 1999 Cancer Research Campaign

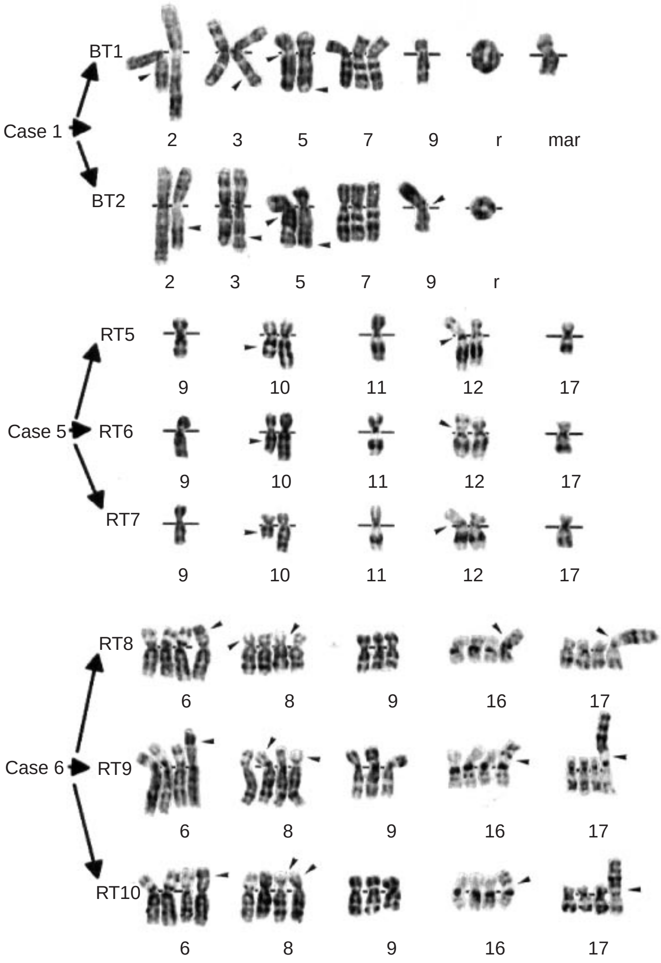

**Figure 2** Partial karyograms from cases 1, 5 and 6. BT, bladder tumour; RT, recurrent tumour. The chromosome indicated by 'mar' represents unidentified marker, and 'r' represents ring chromosomes. Arrowheads indicate breakpoints of abnormalities affecting chromosome 17 in most of the tumours, in spite of the fact that such changes, too, are common in bladder carcinomas, especially in invasive poorly differentiated TCCs (Fujimoto et al, 1992; Grossfeld et al, 1997). In our series, rearrangements of chromosome 17 resulting in loss of the short arm were seen in cases 2, 5 and 6. It is worthy of note, however, that the del(17)(p11) of case 2 was present only in a subclone in the high-grade (G3) lesion (BT7), whereas the two simultaneously occurring low-grade lesions (BT5 and BT6) had no chromosome 17 aberrations. In cases 5 and 6, finally, the lesions, though superficial and of low-grade (G2), were multiple and late recurrences. Our findings thus are in full agreement with the view that alterations of chromosome 9 play a ubiquitous role in the earliest phases of BC tumorigenesis, whereas changes of chromosome 17 signal the transformation to more aggressive tumour behaviour. The crucial gene-level changes behind these chromosomal rearrangements remain uncertain although attractive candidates, e.g. the *TP53* tumour suppressor gene in 17p and the cyclin genes in 9p (Miller et al, 1986; Williamson et al, 1995), have commanded considerable interest and may be pivotal in the tumorigenic process (Reznikoff et al, 1996).

In two of the patients (cases 3 and 4), bladder tumour(s) were associated with a ureteral tumour. Since all the tumours in each case, irrespective of their sites, were cytogenetically related demonstrating their common clonal origin, and since downstream rather than upstream shedding of cancer cells is more likely, the ureteral tumour should be considered the primary one. Epidemiological data are consistent with this conclusion. The presence of an upper urinary tract tumour is associated with a 75% probability of finding tumour(s) also in the bladder, whereas the risk of developing a ureteral tumour following the diagnosis of a bladder tumour is only 4% (Kakizoe et al, 1980; Kenworthy et al, 1996). In addition to the downstream passage of urine, the decreased expression level of E-cadherin by shed cancer cells (Morton et al, 1995) combined with the fact that mucosal inflammation facilitates their subsequent implantation (Rosin et al, 1994), thus provide good explanation for the observed distribution of secondary tumours in BC. The adjuvant use of intravesical BCG is known to reduce the recurrence rate in BC as well as the progression of the disease, mostly by limiting tumour cell motility (Garden et al, 1992). Unfortunately, the attachment of shed tumour cells is not affected by BCG, which may explain the failure of this approach in 20% of the patients (Unyime et al, 1994; Nicol, 1995).

 *© 1999 Cancer Research Campaign*

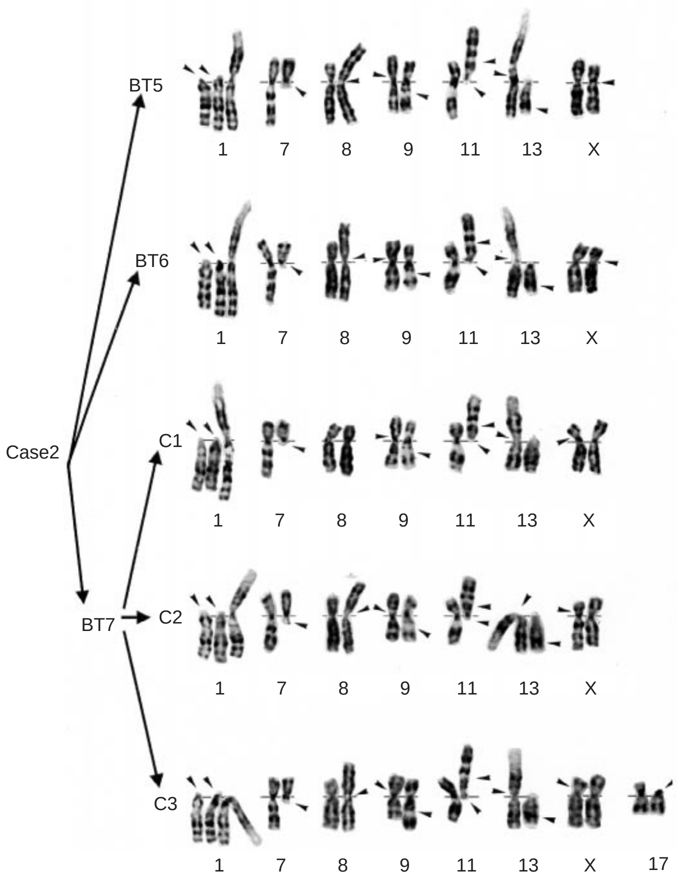

**Figure 3** Partial karyogram from case 2. BT, bladder tumour; C, subclone. Arrowheads indicate breakpoints

Another angle to the present study was to see whether recurrent bladder tumours have the same genetic alterations as do primary ones. The recurrent tumour of case 4 (RT1) developed 3 months after the multiple primary tumours had been removed and at a different site, and yet its karyotype was identical to that of BT12. The absence of additional secondary chromosome abnormalities in the recurrent tumour indicates that the seeding occurred after the clonal evolution had taken place in BT12. In cases 5 and 6, we do not know the karyotype of the original tumour, but again the karyotypic similarity among the multiple recurrent tumours in each case argues strongly for a single-cell origin. Similar to what was seen in the multiple primary tumours, the cytogenetic findings in recurrent BC indicate that the tumorigenic process is monoclonal. A similar conclusion has previously been reached based on the immunohistochemical study of tumour suppressor gene expression in recurrent bladder tumours (Chern et al, 1996). The available cytogenetic information allows no conclusion as to whether tumour cell shedding with subsequent implantation and growth is more likely to occur from the first or later (second) primary tumours, but judging from the clinical and epidemiological data, both situations are common (Breul et al, 1992; Herr et al, 1997).

In spite of the growing understanding of the key role played by tumour cell shedding in the development of second primary bladder carcinomas, other elements in multifocal uroepithelial tumorigenesis remain poorly understood. Sidransky et al (1992) suggested a pathogenetic model based on early intraluminal dissemination of transformed cells followed by the independent growth of each cell, eventually resulting in multiple, clonally related tumours that vary in their pattern of secondarily acquired genetic alterations. Our findings seem to require a certain modification of this view. The extensive cytogenetic similarity observed among all tumours from the same patient, each tumour sharing 5–8 numerical and/or structural chromosome aberrations, suggests, in addition to the common clonal origin, that seeding is a rather late event; such similarity is hardly compatible with an early splitting up of the monoclonal, neoplastic cell population followed by the acquisition of secondary genetic changes.

That intraluminal shedding and implantation of tumour cells is the mechanism responsible for both synchronous and metachronous multifocal BC may be of considerable relevance for therapeutic strategies. Complete endoscopic removal of the primary tumour is often not enough even in the treatment of superficial BC. Additional measures should be directed toward stopping tumour cell seeding and the ability of already implanted tumour cells to divide. This implies more emphasis on intravesical adjuvant therapy in superficial BC, including the use of anti-inflammatory drugs, antagonists of cell adhesion and intravesical chemotherapy

© 1999 Cancer Research Campaign or immunotherapy with BCG. The rationale for avoiding unnecessary trauma to the urothelium during the endoscopic removal of bladder tumours also seems stronger. The practice of taking random bladder biopsies or removing a benign prostate at the time of bladder tumour surgery seems illogical in this perspective and should probably be discontinued.

### ACKNOWLEDGEMENTS

This work was supported by grants from the Swedish Society for Medical Research, the Tatjana and Jacob Kamras Research Fund, the Medical College at Lund University, and the Swedish and Norwegian Cancer Societies. We thank Dr Anders Ek, Birgitta Lundgren and Liselott Bjurell from the Department of Urology, Helsingborg Hospital, for their assistance in collecting the tumour samples.

### REFERENCES

Badalament RA and Schervish EW (1996) Bladder cancer, current diagnostic methods and treatment options. *Postgrad Med* **100**: 217–230

Breul J, Block T, Breidenbach H and Hartung R (1992) Implantation metastasis after a suprapubic catheter in a case of bladder cancer. *Eur Urol* **22**: 86–88

Chern HD, Becich MJ, Persad RA, Romkes M, Smith P, Collins C, Li YH and Branch RA (1996) Clonal analysis of human recurrent superficial bladder cancer by immunohistochemistry of *p53* and retinoblastoma proteins. *J Urol* **156**: 1846–1849

Fadl-Elmula I, Gorunova L, Lundgren R, Mandahl N, Forsby N, Mitelman F and Heim S (1998) Chromosomal abnormalities in two bladder carcinomas with secondary squamous cell differentiation. *Cancer Genet Cytogenet* **102**: 125–130

Fitzpatrick JM, West AB and Butler MR (1986) Superficial bladder tumors (stage pTa grade 1 and 2). The importance of recurrence pattern following initial resection. *J Urol* **135**: 920–922

Fujimoto K, Yamada Y, Okajima E, Kakizoe T, Sasaki H, Sugimura T and Terada M (1992) Frequent association of *p53* gene mutation in invasive bladder cancer. *Cancer Res* **52**: 1393–1398

Garden RJ, Liu BC and Redwood SM (1992) Bacillus Calmette-Guerin (BCG) abrogates in vitro invasion and motility of human bladder tumor cell via fibronectin interactions. *J Urol* **148**: 900–950

Gibas Z and Gibas L (1997) Cytogenetics of bladder cancer. *Cancer Genet Cytogenet* **95**: 108–115

Grossfeld GD, Ginsberg DA, Stein JP, Bochner BH, Esrig D, Groshen S, Dunn M, Nichols PW, Taylor CR, Skinner DG and Cote RJ (1997) Thrombospondin-1 expression in bladder cancer: association with *TP53* alterations, tumor angiogenesis, and tumor progression. *J Natl Cancer Inst* **89**: 219–227

Habuchi T, Takahashi R, Yamada H, Kakehi Y, Sugiyama T and Yoshida O (1993) Metachronous multifocal development of urothelial cancers by intraluminal seeding. *Lancet* **342**: 1087–1088

Harris AL and Neal DE (1992) Bladder cancer, field versus clonal origin. *N Engl J Med* **326**: 759–761

Heim S (1992) Is cancer cytogenetics reducible to the molecular genetics of cancer cells? *Genes Chromosomes Cancer* **5**: 188–196

Heney NM, Proppe K, Prout GR, Griffin PP and Shipley WU (1983) Invasive bladder cancer: tumor configuration, lymphatic invasion and survival. *J Urol* **130**: 895–902

Heney NM, Ahmed S and Flanagan MJ (1983) Superficial bladder cancer. Progression and recurrence. *J Urol* **130**: 1083–1086

Herr HW (1997) Natural history of superficial bladder cancer: 10- to 20-year follow-up of treated patients. *World J Urol* **15**: 84–88

ISCN (1995) *An International System for Human Cytogenetic Nomenclature.* Mitelman F (ed.). S Karger: Basel

Kakizoe T, Fujita J, Murase T, Matsumotoko K and Kishi K (1980) Transitional cell carcinoma of the bladder in patients with renal pelvis and ureteral cancer. *J Urol* **124**: 17–19

Kenworthy P, Tanguay S and Dinney CP (1996) The risk of upper tract recurrence following cystectomy in patients with transitional cell carcinoma involving the distal ureter. *J Urol* **155**: 501–504

Kiemeney LA, Witjes JA, Heijbroek RP, Koper NP, Verbeek AL and Debruyne FM (1993) Predictability of recurrent and progressive disease in individual patients with primary superficial bladder cancer. *J Urol* **150**: 60–64

Lynch CF and Cohen MB (1995) Urinary system. *Cancer* **75**: 316–345

Miller C, Mohandas T, Wolf D, Prokocimer M, Rotter V and Koeffler HP (1986) Human *TP53* gene localized to short arm of chromosome 17. *Nature* **319**: 783–784

Miyao N, Tsai YC, Lerner SP, Olumi AF, Spruk CH, Gonzalez-Zulueta M, Nichols PW, Skinner DG and Jones PA (1993) Role of chromosome 9 in human bladder cancer. *Cancer Res* **53**: 4066–4070

Morton RA, Ewing CM, Watkins JJ and Isaac WB (1995) The E-cadherin cell adhesion pathway in urologic malignancies. *World J Urol* **13**: 364–372

Nicol D (1995) BCG in bladder cancer: a warning. *Aust NZ J Med* **25**: 275–282

Parker SL, Tong T, Bolden S and Wingo PA (1996) Cancer statistics. *Ca Cancer J Clin* **46**: 5–27

Prout GR, Barton, Griffin PP and Friedell GH (1992) Treated history of noninvasive grade 1 transitional cell carcinoma. The National Bladder Group. *J Urol* **148**: 1413–1419

Reznikoff CA, Belair CD, Yeager TR, Savelieva E, Blelloch RH, Puthenveettil JA and Cuthill SA (1996) Molecular genetic model of human bladder cancer pathogenesis. *Semin Oncol* **23**: 571–584

Richie JP, Shipley WU and Yagoda A (1989) Cancer of the bladder. In: *Cancer Principles and Practice of Oncology*, De Vita VT, Helman S and Rosenberg SA (eds), pp. 1008–1022. JB Lippincctt: Philadelphia

Rosin MP, Anwar WA and Ward AJ (1994) Inflammation, chromosomal instability, and cancer: the schistosomiasis model. *Cancer Res* **54**: 1929–1933

Schmitz-Dräger BJ, Jankevicius F and Ackermann R (1996). Molecular biology of dissemination in bladder cancer laboratory findings and clinical significance. *World J Urol* **14**: 190–196

Sidransky D, Frost P, Von-Eschenbach A, Oyasu R, Preisinger AC and Vogelstein B (1992) Clonal origin of bladder cancer. *N Eng J Med* **326**: 737–740

Thompson RA, Campbell EW, Kramer HC, Jacobs SC and Naslund MJ (1993) Late invasive recurrence despite long-term survival for superficial bladder cancer. *J Urol* **149**: 1010–1011

Union International Contre le Cancer (1978) *TNM Classification of Malignant Tumors*. Union International Contre le Cancer: Geneva

Unyime ON and Donald LL (1994) Therapy of superficial cancer. *Curr Opin Urol* **4**: 275–280

Williamson MP, Elder PA, Shaw ME, Devlin J and Knowles MA (1995) *P16 (CDKN2)* is a major deletion target at 9p21 in bladder cancer. *Hum Mol Genet* **4**: 1569–1577

Wolf H and Hojgaard K (1982) Urothelial dysplasia concomitant with bladder tumors as a determinant factor for future new occurrences. *Lancet* **2**: 134–140

World Health Organization (1973) *Histological Typing of Urinary Bladder Tumors.* International Histological Classification of Tumors: Geneva

Xu X, Stower MJ, Reid NI and Garner RC (1996) Burns PA Molecular screening of multifocal transitional cell carcinomas of the bladder using *TP53* mutations as biomarkers. *Clin Cancer Res* **2**: 1795–1800

Yao A and Rubin HA (1994) Critical test of the role of population density in producing transformation. *Proc Natl Acad Sci USA* **91**: 7712–7716

 *© 1999 Cancer Research Campaign*
